# Health and social care of home-dwelling frail older adults in Switzerland: a mixed methods study

**DOI:** 10.1186/s12877-022-03552-z

**Published:** 2022-11-15

**Authors:** Olivia Yip, Suzanne Dhaini, Jan Esser, Flaka Siqeca, Maria Jose Mendieta, Evelyn Huber, Andreas Zeller, Sabina De Geest, Mieke Deschodt, Franziska Zúñiga, Leah L. Zullig, Heather A. King, the INSPIRE consortium, Pia Urfer, Penelope Vounatsou, Katrina Obas, Matthias Briel, Matthias Schwenkglenks, Carlos Quinto, Eva Blozik

**Affiliations:** 1 Institute of Nursing Science, Department of Public Health, University of Basel, Bernoullistrasse 28, 4056 Basel, Switzerland; 2 Department of Neurosurgery, Medical Center - University of Freiburg, Freiburg, Germany; 3 Academic Center for Nursing and Midwifery, Department of Public Health and Primary Care, KU Leuven, Leuven, Belgium; 4 Institute of Nursing, School of Health Professions, ZHAW Zurich University of Applied Sciences, Winterthur, Switzerland; 5 Centre for Primary Health Care, University of Basel, Basel, Switzerland; 6 Gerontology and Geriatrics, Department of Public Health and Primary Care, KU Leuven, Leuven, Belgium; 7 Competence Center of Nursing, University Hospitals Leuven, Leuven, Belgium; 8 Department of Population Health Sciences, Duke University School of Medicine, Durham, NC USA; 9 Center of Innovation to Accelerate Discovery and Practice Transformation, Durham Veterans Affairs Health Care System, Durham, NC USA; 10 Division of General Internal Medicine, Department of Medicine, Duke University School of Medicine, Durham, NC USA

**Keywords:** Delivery of healthcare, integrated, Community-care, Frail elderly, Social support, Formal and informal care

## Abstract

**Background:**

Home-dwelling frail older adults are often faced with multimorbidity and complex care needs, requiring health and social care systems that support frail older adults to age in place. The objective of this paper was to investigate the types of formal health and social care as well as informal care and social support used by home-dwelling frail older adults; whether they perceive their support as sufficient; and their experience with and preferences for care and support.

**Methods:**

Using an explanatory sequential mixed methods design, we first conducted a secondary analysis of a subset of cross-sectional data from the ImplemeNtation of a community-baSed care Program for home dwelling senIoR citizEns (INSPIRE) population survey using descriptive analysis. Subsequently, we analyzed existing data from interviews in the parent study to help explain the survey results using applied thematic analysis. Results were organized according to adapted domains and concepts of the SELFIE framework and integrated via a joint display table.

**Results:**

Of the parent population survey respondents, 2314 older adults indicating frailty were included in the quantitative arm of this study. Interview data was included from 7 older adults who indicated frailty. Support from health and social, formal and informal caregivers is diverse and anticipated to increase (e.g., for ‘care and assistance at home’ and ‘meal services’). Informal caregivers fulfilled various roles and while some older adults strongly relied on them for support, others feared burdening them. Most participants (93.5%) perceived their overall support to meet their needs; however, findings suggest areas (e.g., assessment of overall needs) which merit attention to optimize future care.

**Conclusions:**

Given the anticipated demand for future care and support, we recommend efforts to prevent fragmentation between health and social as well as formal and informal care.

**Supplementary Information:**

The online version contains supplementary material available at 10.1186/s12877-022-03552-z.

## Background

Aging in place is a common goal for home-dwelling older adults [1], requiring health and social care systems that support the older person to continue to live at home [2–4]. However, living at home independently can become a major challenge for frail older adults [5], who are often faced with functional limitations, multimorbidity and complex care needs [6, 7]. They depend on health and social care and support, which may involve multiple formal (e.g., professionals, care organizations) and informal caregivers (e.g., family members, neighbors) [8–10]. In the community setting, care and support for frail older adults is often fragmented and uncoordinated, leaving them at risk for negative health outcomes [11, 12]. Care should ideally be based on a formal assessment and tailored to older adults’ needs and preferences, as well as integrated [13], whereby interprofessional collaboration and coordination between all relevant caregivers is leveraged to support frail older adults to age in place [14–16].

To help the aging population remain living at home despite their high care needs, and to avoid costly institutional care, there will be an increasing need for both health and social care from formal and informal caregivers [8, 9]. Health care services include “acute, chronic, preventive, restorative and rehabilitative care”, delivered by various providers [17], while social care includes a wide variety of services which provide “physical, emotional and social support to help people live their lives” [18]. Formal care at home includes health or social services provided by (mostly) paid and trained professionals, such as home care nurses or household services [19]. Informal care occurs when care is provided without payment or formal training, typically provided by a spouse, children, family and friends or neighbours [10, 19]. Informal care includes assisting with activities of daily living (e.g., bathing and eating), or instrumental (e.g., transportation and finances), assisting with medical or nursing tasks, or providing emotional support [10]. Due to the challenges which result from fragmentation between health and social care, integration has been widely promoted [20–22]. Both formal and informal care are well-researched; however, only more recently have researchers and policy-makers considered the intersection of these two approaches to caregiving for home-dwelling frail older adults [19, 23–26], an area of growing interest [24]. One study suggests that while non-frail older adults used informal care often as a substitute for formal care, frail older adults appeared to use both in compliment [8]. Although formal and informal caregivers should ideally work together, this is another gap recognized in community-based care for older persons [19, 24]. Bridging these “problematic divides” between health and social as well as formal and informal care is important when moving towards care integration [16, 27], i.e., optimally collaborating and communicating on aspects such as shared decision-making and care planning [28, 29], and all caregivers fulfilling their key roles in supporting the older person according to their needs and preferences [5, 16]. Therefore, when planning future care services for the aging population, it is helpful to first understand the specific sources and contributions from formal health and social care as well as informal care and social support used and preferred in future by the frail population. Such insight can help allocate resources and organize services which are coordinated and delivered around the needs and preferences of older adults [4], as well as identify and collaborate with local stakeholders who will become increasingly involved in caring for older adults in the community.

The various individuals involved in providing care and support to help meet the needs of home-dwelling frail older adults have often been studied as “care networks” [5, 8, 26, 30–32], or more recently as “care convoys” [23, 33]. Researchers identified the diversity within the structure of care networks or convoys [26], reporting multiple different combinations of informal and formal care use [33, 34], and occasionally explored whether frail older adults perceive their care and support to meet their needs [5, 33]. As shown by Verver et al.’s (2018) study, 33.7% of frail individuals living independently did not have the care and support that they needed e.g., social contacts or domestic help, even though they had more formal care providers and were more likely to have informal care providers involved than their non-frail counterparts [5]. Lambotte et al. (2020) also noted that a frail person’s satisfaction with his/her care convoy did not necessarily mean they had sufficient help, and vice versa [33]. Although these needs are bound to increase over time and would likely need to be iteratively re-assessed, it is important to understand in what ways the care and support of frail older adults are meeting their needs and to detect any gaps. Identifying these gaps and determining how to engage and support those living with unmet needs should be a priority given the risk and vulnerability associated with frailty [35, 36].

The present study is part of the larger INSPIRE (ImplemeNtation of a community-baSed care Program for home dwelling senIoR citizEns) parent study taking place in one canton, Basel-Landschaft (BL), an area in the North-western part of Switzerland. A cantonal care law enforced in 2018 ensued that older adults living at home will have access to a new information and advice center (IAC) for advice related to care and nursing in old age, as well as an assessment of needs, and either mediation of care or potential nursing home referral [37, 38]. The INSPIRE project aims to develop, implement, and evaluate an integrated care model for these IACs [38]. During the development phase, a contextual analysis was conducted which included a population survey [39] followed by interviews with older adults [40] to create an IAC care model which was suited to local health and social needs and preferences. More information on the parent study can be found elsewhere [38–40]. Using a quantitative approach, we investigated the type and frequency of formal health and social care as well as informal care and social support that frail older adults are currently using and their future preferences, and to what extent the older adults perceive their support in place meets their current needs. Subsequently, in the qualitative arm, we aimed to gather a more in-depth understanding of their experience and preferences with their care and support, and explore appearance of integrated care concepts (e.g., presence of a named coordinator or multi-disciplinary care team). Using a mixed methods approach, we unified this data to explore the types of care and support used by home-dwelling frail older adults as well as their experience and future preferences.

## Methods

### Study design

**Fig. 1:**
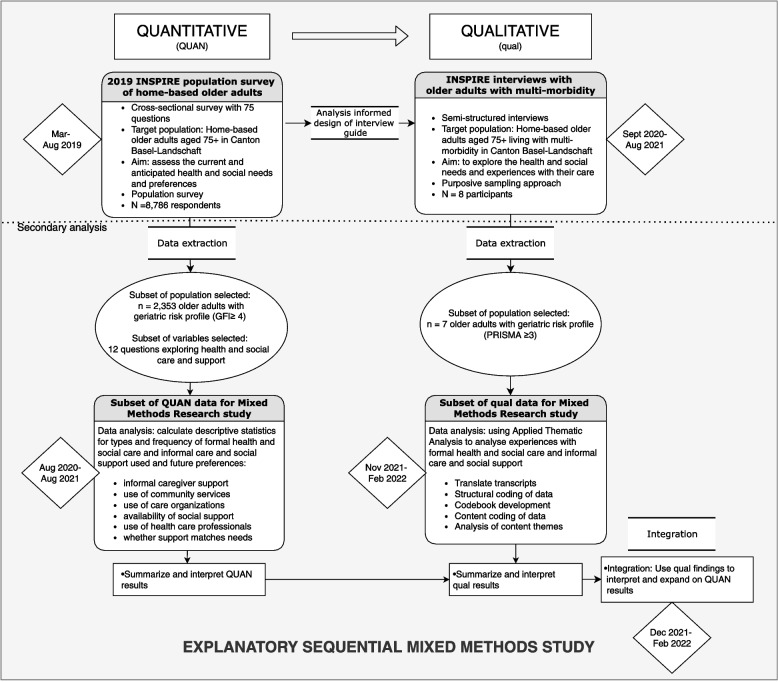
Design of the explanatory sequential mixed methods study of frail older adults’ health and social care and support. GFI: Groningen Frailty Indicator (screening tool); PRISMA-7: Program of Research to Integrate Services for the Maintenance of Autonomy (screening tool); Note. The research method which was conducted first is denoted in capitalized letters (QUAN)

We used an explanatory sequential mixed methods design (Fig. 1) [41]. First, we conducted secondary analysis on a subset of data from the cross-sectional INSPIRE population survey [39] (which was part of the parent study). To help explain and expand on these results [41], we used data from interviews with older adults in the INSPIRE parent study [40] (which used Interpretive Description [42]).

### Phase 1: Quantitative

#### Sample

The current study included a sub-sample of frail older adults from the INSPIRE population survey in the parent study. In the population survey, the Groningen Frailty Indicator (GFI) [43, 44] was used to assess the geriatric risk profile of participants (*N* = 8786; response rate = 30.7%), as the GFI considers frailty to be a multidimensional construct which includes physical, psychological, social, and cognitive domains [44, 45]. Using this broader definition to measure frailty and determine those at risk of institutionalization (for example) based on their geriatric risk profile implies that this sub-sample represents a group experiencing frailty in more diverse areas than solely biomedical indicators [46]. Regardless of whether participants answered all 15 GFI questions, the quantitative arm of the present study included only those with a GFI score of 4 or more (i.e., considered frail), resulting in a sample of *n* = 2314 frail older adults.

#### Variables and measurements

We selected variables from the INSPIRE population survey [39] (Additional file 1). Except for sample characteristics, variables are presented according to adapted domains (i.e., Individual with multi-morbidity and their environment, Workforce, and Leadership and governance) and concepts (e.g., needs, social network, use of community services, use of transportation services, informal caregiver support, use of health care professionals, use of care organizations, named coordinator, multi-disciplinary team, individualized care planning) of the SELFIE framework, to stay consistent with the organization of results [28]. The SELFIE (Sustainable intEgrated chronic care modeLs for multi-morbidity: delivery, Financing, and performancE) framework has been commonly used in European studies to support development, description, implementation and evaluation of integrated care initiatives, and emphasizes important concepts within integrated care, such as presence of a care coordinator [28].

##### Socio-demographic information and frailty status

Participants’ socio-demographic characteristics were summarized, including age (year of birth), gender, education level, monthly household income, and household members. Additionally, the geriatric risk profile was determined by the individual’s GFI score. The GFI is a reliable and valid 15-item instrument for frailty screening [43–45]. A GFI score of greater than four indicates frailty [44].

##### Individual with multi-morbidity and their environment

Needs (met/unmet): one investigator-designed question with a “yes/no” option measured whether their support received in everyday life meets their needs.

Social network: availability of social support was assessed using the validated German version of the Brief Social Support Scale (BS6) [47]. There are six items to be rated on a 4-point Likert scale, divided by tangible support (e.g., how often is there someone available to prepare your meals if you are unable to do it yourself) and emotional-informational support (e.g., how often is there someone available who understands your problems). Responses were dichotomized (“never” versus “sometimes”, “often”, and “always”) for the analysis.

Use of community services (formal care) was measured through two questions to capture the types of community services (e.g., meal services, transportation services) needed or used in 2018 and services which would be considered if they become more in need of help in the future.

##### Workforce domain

Informal caregiver support in everyday life and preferences for future (if they become more in need of help) was captured through two questions designed by the research team which contained different options for sources of support (i.e., relatives of the same age [e.g., spouse]; younger family members; friends and neighbours; or none).

Whether participants were caregivers themselves was determined through one binary question designed by the research team which asked if they looked after, cared for or supported another person (i.e., children, older persons, or someone with a disability).

Use of health care professionals (formal care) in 2018 was measured using three questions which examined frequency of General Practitioner (GP) visits, specialist visits, and an open-ended question for other medical services used.

Use of care organizations (formal care) was measured through two questions to capture the current care organizations providing regular support in everyday life and care organizations preferred in future if they become more in need.

#### Statistical methods

Consistent with the INSPIRE population survey in the parent study [39], selected socio-demographic variables were descriptively analysed and reported as frequencies, percentages, medians, and interquartile range (IQR) to describe the sample of survey participants. We also included a description of household members to support interpretation of care use. Frequencies were reported for dichotomized or categorical survey variables. However, many of the survey questions were set-up to allow each participant to give multiple responses per question. For these questions with multiple response options, the proportion of respondents accounted for by each item were reported. When respondents provided inconsistent answers (i.e., provided a contradictory answer to the question), we excluded them from analysis within the respective survey domain (Additional file 2).

To analyze three of the survey variables, use of community services, informal caregiver support, and care organizations, we first dichotomized the responses to indicate whether each type of support was currently used or not. We then selected only those who provided an answer for both current use and future preferences in order to compare them, excluding those who did not provide an answer for future preferences. Following principles of a sensitivity analysis, we analyzed our data with and without the excluded individuals to confirm that the results were not impacted. Finally, we separately analyzed those who did not currently use the support, but provided data on future preferences, to further inform future predictions.

Missing data was assumed to be missing at random for all questions (except formal care services) and therefore excluded, but amount of missing data was reported throughout.

A brief sensitivity analysis was performed to address how we calculated the GFI score. We calculated the total GFI score by summing each score of “0” or “1” to the 15 items that comprise the GFI. We categorized anyone with a GFI score greater than or equal to four as frail, regardless of how many total GFI-items they answered, to avoid losing power. We analyzed to see if the results would have been different had we required an answer to all 15 questions, but values remained consistent.

Analyses were performed using IBM SPSS Statistics (Version 26).

### Phase 2: Qualitative

#### Sample

Using purposive sampling, semi-structured interviews were conducted in the INSPIRE parent study between September 2020 and August 2021 with eight home-dwelling older adults with multimorbidity, defined as the occurrence of two or more chronic diseases [48]. Furthermore, individuals had to be using health services provided by at least two care organizations, or three or more different health services provided by one organization [40]. In the current study, we included interview data from individuals who indicated frailty based on the PRISMA-7 frailty screening assessment [49] and considered the sample size to be adequate based on information power (*n* = 7), i.e., whether the sample size was sufficient to contribute knowledge in response to the research question, determined by aspects such as the study aim, sample specificity and whether the study is theoretically-informed [50]. The PRISMA-7 includes seven dichotomous questions, and was completed by the researchers according to the participants’ responses and interactions with them during the interviews, as well as the demographic data collected. The lead author (OY) screened the original interview participants’ anonymous PRISMA-7 scores to identify eligible participants (i.e., scoring ≥3 which is indicative of frailty [49]) for the present study. The PRISMA-7 assessment instead of the GFI was used in the interviews to reduce burden on the participants, given the majority of the information collected through the PRISMA-7 was easily observable by the researchers or already embedded within the interview questions.

#### Instruments

The interview guide (see Additional file 3) was developed in the parent study to build on the INSPIRE population survey findings and further explore older adults’ health and social needs and experience of their care and support. We incorporated additional concepts which are key to integrated care (e.g., informal caregiver support, a named coordinator) from literature such as the SELFIE framework into the interview guide, to get a sense of the presence of these concepts in their current care [28].

#### Analysis

Before the anonymous interview transcripts were translated into English (as the lead author was not fluent in German), the original German transcripts were cleaned from all filler words and Swiss-German nuances were translated to High-German. Validation of content was performed only for discrepancies between the two German dialects, or between the German and English languages. Applied Thematic Analysis was then used to analyse the transcripts [51]. First, the lead author (OY) created a research map to establish structural codes based on the domains in the interview guide. Next, the lead author performed structural coding on the data using NVivo [52], to organize the data by the structural codes (i.e., according to the concepts from the interview guide or discussion of the concept during the interview), which led to a coding report for each structural code [53]. Thereafter, content coding was performed, to analyse the data within each structural coding report [53]. A separate codebook was created for each structural code to contain all content codes. For each structural coding report, a memo was written to describe the content codes and help to derive themes.

#### Techniques to enhance trustworthiness

Given the nature of this study, we considered the following techniques to enhance trustworthiness [54].

##### Context

The original interviewer made the context of participants’ responses fully available through providing access to the transcripts and a thorough written description of the interview setting. Consistent with participants’ preferences, most interviews took place in the individual homes of the participants in Canton BL, with one interview taking place at a nursing home during a short stay. Some participants had a family member present during the interview, while others were alone. In most cases, there were two INSPIRE interviewers present.

##### Rigor

In aiming for consensus, initial results were presented to the larger research team and original interviewers throughout the analysis, to gather feedback and input based on their methodological, clinical and local expertise. A precise codebook was also developed. To maintain an audit trail, all notes, memos, changes to the codebooks, and analytical documents which were prepared during the study have been archived.

#### Data integration

For analysis, we organized the quantitative and qualitative findings according to the adapted domains and concepts of the same theoretical framework, SELFIE [28]. At the interpretation and reporting phase, the quantitative and qualitative findings were first integrated through a joint display table and later synthesized via weaving (i.e., written up together on a concept-by-concept basis) [41, 55, 56]. With the goal and principles of conducting a value-adding qualitative analysis in mind [57], the original interview findings from the INSPIRE parent study were occasionally included in the results of this paper, to help explain the survey findings and allow for discourse and reflections targeting integrated care.

#### Ethical consideration

For ethical review, the population survey was submitted to the Ethikkommission Nordwest- und Zentralschweiz (EKNZ) in Switzerland, BASEC Nr Req-2019-00131. It did not meet the definition of a research project requiring further review as per the Human Research Act ART.2, and was able to move forward as it met the general ethical principles for research involving humans (cf. Art. 51 para. 2 Human Research Act). Data collection for the interviews was approved by the EKNZ under Project ID: 2020–01755. To conduct the mixed methods study, a clarification of responsibility was submitted to the EKNZ, however it did not require further review (Project ID: Req-2021-00170).

## Results

### Survey participants

In total, 2314 INSPIRE population survey participants were eligible for this study, but not all participants responded to each survey question (Additional file 2). There were 594 participants who provided a response to all survey questions discussed in this paper. Participant ages ranged from 75 to 107 years with 60.3% being female (Table 1). The median GFI score was 5.

**Table 1:** Participant Characteristics of the INSPIRE Population Survey, for Frail Respondents (*n* = 2314)

Characteristics	% (*n*)	Median [IQR]
Age		83 [79–87]
75–79	27.1% (628)	
80–84	33.4% (774)	
85–89	26.0% (601)	
90–94	10.1% (234)	
95–99+	3.3% (77)	
Female gender	60.3% (1385)	
Education		
No degree	1.9% (43)	
Elementary school	18.6% (421)	
Vocational training ^a^	48.1% (1091)	
High School ^b^	4.9% (112)	
University ^c^	21.5% (488)	
Other	4.9% (112)	
Household Income (monthly) ^d, e^		
< 3000 CHF	14.4% (315)	
3001–6000 CHF	39.9% (873)	
> 6000 CHF	29.5% (644)	
Do not know	1.8% (40)	
Do not wish to answer	14.4% (314)	
Household members ^f^		
Live alone	44.7% (1026)	
Live with spouse/partner	51.7% (1188)	
Live with siblings	0.2% (5)	
Live with adult children	3.2% (73)	
Live with other^g^	3.4% (79)	
GFI Score ^h^		5.0 [4–6]

^a^indicates completion of an apprenticeship (e.g., hairdressing; electrician)

^b^High School = a preparatory step for University

^c^University of Applied Sciences or University

^d^Missing data: household income (*n* = 128; 5.5%)

^e^a monthly income of 2459CHF was used as the threshold to consider a person at-risk-of-poverty in Switzerland for 2018 for a single person [58]

^f^Multiple responses possible, therefore percent of respondents shown

^g^combined response for other adults, professional help or other

^h^possible GFI score range: 4–15 (as minimum GFI score was ≥4 to be included in the study)

### Interview participants

The characteristics of the seven interviewees in this study are reported as part of Esser et al.’s (2022) study [40]. In summary, four of the interview participants were men; most were single and living alone; had an education level of vocational training or higher; and had a range of four to eight chronic diseases. The mean PRISMA-7 score was 5.6 (note: the possible scores can range from 0 to 7, where a minimum PRISMA-7 score was ≥3 to be included in the study as this indicates frailty [49]).

The integration of quantitative and qualitative findings through a joint display table (Additional file 4) informed the presentation of results below.

#### Individual with multi-morbidity and their environment

##### Needs and preferences

Overall, most survey respondents (94%) perceived that the support they receive in everyday life meets their needs (Table 2). Interviewees described care and support from multiple services or professionals as going well, such as physiotherapy, social, or home care services, the latter described by one as “impeccable” (M3). As also acknowledged by Esser et al. (2022), all discussed their strong desire to continue living at home [40], “for me it has always been my goal in life to avoid the nursing home” (M4). Interviewees were pleased when they received personal care which helps them to meet their goals “it’s nice when you find the person who can treat you individually. That is a gift” (F4), but also recognized that personal care “is not easy at all, because every person is special in their own way” (F4). Interestingly, while a lack of continuity was occasionally discussed as challenging, others experienced this not to be problematic. One participant pointed out the gaps she sees for others “But where I actually have a big problem … is that I know people who are in a similar situation to mine, who are alone and have no one to help them” (F2). Interviewees mostly felt that an overall assessment of their needs was often not performed but two could see this would be beneficial, while a few believed this had been performed by their home care service.

**Table 2:** Concepts related to care and support of frail older adults in 2018 and preferences for future, mapped to adapted domains of the SELFIE framework (*N* = 2314 unique participants)

	In 2018% (*n*)	Future preferences % (*n*)
**INDIVIDUAL WITH MULTI-MORBIDITY AND THEIR ENVIRONMENT**		
**Needs**		
Support matches needs ^a^	93.5% (1947/2083)	
**Social network** (‘Sometimes’, ‘often’ or ‘always’) ^b^		
Someone who will take you to the doctor if necessary	61% (1293/2114)	
Someone who prepares food for you when you’re not able to	44% (881/1997)	
Someone to help you with your day-to-day work when you’re sick	70% (1408/2014)	
Someone who can give you good advice in difficult situations	76% (1556/2046)	
Someone you can trust or talk to about personal problems	88% (1883/2132)	
Someone who understands your problems	88% (1809/2053)	
**Use of community services**^c, d, e, j^		
Help with the housework	57% (650/1132)	81% (919/1132)
Care and assistance at home	35% (398/1132)	86% (978/1132)
Meal service	13% (143/1132)	49% (550/1132)
Elderly day care centre^f^	4% (41/1132)	6% (62/1132)
Apartment for older adults	3% (36/1132)	22% (251/1132)
Short stays in a Nursing home	3% (36/1132)	22% (246/1132)
Care centre with nighttime services	1% (16/1132)	2% (25/1132)
Other (e.g., Hospital, cleaning)	14% (157/1132)	3% (37/1132)
I do not know		13% (147/1132)
**Transport**^c, d, e, j^		
Transportation and assistance services (e.g., to doctor’s office, shopping)	24% (274/1132)	51% (572/1132)
**DOMAIN: WORKFORCE**		
**Informal caregiver support**^c, e, j^		
Family members of the same age (e.g., spouse, partner)	56% (863/1542)	56% (870/1542)
Younger family members (e.g., children, grandchildren)	55% (845/1542)	67% (1033/1542)
Friends and neighbours	23% (350/1542)	30% (454/1542)
**Use of health care professionals**^d^		
Physiotherapy ^c, e^	45% (505/1132)	43% (486/1132)
GP visits^g^		
0 visits	2% (46/2163)	
1–6 visits	60% (1307/2163)	
7–10+ visits	37% (810/2163)	
Specialist visits^g^		
0 visits	16% (328/2045)	
1–6 visits	67% (1376/2045)	
7–10+ visits	17% (341/2045)	
Other medical services (e.g., dentist, eye doctor)	59% (1368/2314)	
**Use of care organizations**^c, h, d, e^		
Private help (self-payment)	47% (466/996)	43% (425/996)
Non-profit aid (e.g., home care support)	42% (417/996)	86% (855/996)
Pro Senectute (a non-profit foundation serving older adults)	9% (92/996)	26% (261/996)
Red Cross Baselland	6% (63/996)	10% (100/996)
Associations ^i^	3% (27/996)	6% (58/996)
Nursing home		23% (227/996)
Other (e.g., help with cleaning)	20% (195/996)	7% (74/996)

Note. Participants had the opportunity to express their use of health and social care and support across multiple domains. The content domains are not mutually exclusive. Grey boxes indicate that the answer option was not available

^a^Missing responses = 10%

^b^Missing responses: a) *n* = 200, 9% b) *n* = 317, 14% c) *n* = 300, 13% d) *n* = 268, 12% e) *n* = 182, 8% f), *n* = 261, 11%

^c^% of responses = The proportion of the respondents accounted for by this category due to multiple responses possible. To record responses to questions where respondent can give more than one answer

^d^considered to be “Formal care” in this paper

^e^denominator was restricted to only respondents who answered both questions for current use and future preferences

^f^day care center (e.g., providing advice, support, care and integration)

^g^missing responses – GP: 7%; Specialist: 12%

^h^missing responses – 56% who did not respond and/or are not receiving help from an organization

^i^Associations: Combined values for Alzheimer’s association, Parkinson’s association and Diabetes association

^j^older adults who responded that they did not need support were reported in Additional file 5

##### Social network

Availability of social support was lower for the ‘tangible’ support items (e.g., having someone to take them to the doctor) ranging from 44 to 70%, and higher for ‘emotional-informational’ items (e.g., having someone to talk to about their personal problems) (range: 76–88%). Interviewees gave examples of receiving both types of tangible and emotional-informational support (Additional file 4). Moreover, it was discussed that sharing experiences and maintaining a social network was important, but it was easier for some than others, “I have a lot of visitors. And I didn’t know that when you look after your friends that it would come back one day. And it does come back” (F2).

##### Use of community services (formal care)

Use of at least one community service was 58% (Table 2); meanwhile a large proportion (42%) reported that they did not need help in 2018 (Additional file 2). Of those using services, most participants (57%) appeared to be using multiple different services. Among current and future service users, the highest frequency of responses for current use was for help with the housework (57%). Care and assistance at home was the most preferred choice in future (86%), even for those who did not use current services in 2018 (86%; Additional file 5). Interviewees often described their need for support with housework and home care, which also benefits them in additional ways, such as motivating them “if they didn’t come tomorrow … I don’t get dressed or I don’t wake up and I don’t open shutters” (F2). Most types of service use doubled between 2018 and future preferences. Interviewees described various purposes for transportation services.

There was currently a low use of meal services (13%) despite that many reported they never had someone to help prepare food for them when they are not able to in the Brief Social Support Scale (BS6) questions, yet strong demand for meal services in future (Table 2 and Additional file 5).

#### Workforce domain of the SELFIE framework

##### Informal caregiver support

Three-quarters of the survey respondents indicated having at least one source of informal care (Additional file 2). Of those receiving informal care, 71% of respondents relied on one source while 29% relied on two or more sources listed, and interviewees likewise described diverse informal care sources. Support from family members of the same age (56%) will continue to be sought in future by those who will still want informal care, but the largest increase was seen for younger family members. Of those who did not use informal care in 2018, 55% preferred younger family members provide them support in the future if needed, with many also desiring support from others e.g., neighbours. As similarly identified by Esser et al. (2022) the roles of informal caregivers were diverse, with neighbors helping with mail, transport or checking-in on them, while relatives provided the widest support (e.g., communicating with formal caregivers, arranging services and appointments, cleaning, cooking, accompanying to appointments, managing bank payments) [40]. Some interviewees indicated complete reliance on informal caregivers, such as “so if I didn’t have her [the daughter], and the son-in-law, then we would be, I would be lost” (M2). Two described how their daughters help encourage their mobility or independence, and one interviewee described his appreciation for his daughter emotionally, “sometimes when she comes and when she leaves I have tears. Because she always helps me so much and does everything for me” (M2). Yet on the other hand, some interviewees stressed the importance of avoiding burden on their family “I’m very happy that I have family ( …) but I didn’t want to involve them ( …) in my care and in my dependence... I want my children to be free of this burden” (M4). Meanwhile, M4 also implied that informal care would come first before being dependent on help. With respect to their own role in informal care, one-fifth (20%) of the survey participants reported also caring for someone else.

##### Use of health care professionals (formal care)

Participants were asked how often they visited their family doctor and a specialist in 2018. The majority (60%) had 1–6 visits to the family doctor and a large proportion (37%) had 7–10+ visits. During the interviews, GPs appeared quite central in the older adults’ discussions about their care, and were often reported as the main contact person for their health questions, as also seen in Esser et al. (2022) [40]. Likewise, for specialist visits, the majority (67%) also had 1–6 visits, with a decent share having more than 7 visits. Thirty percent indicated they used physiotherapy services in the past year, and many (59%) provided at least one response when asked in an open-ended question about any other medical services they used in 2018, such as a dentist or eye doctor. All interviewees used physiotherapy, who on top of mobility support and exercises, could provide unique value in helping them to practically cope with their situation and for example, taking them for walks in the forest where they would otherwise be restricted to get to. This same interviewee mentioned her appreciation for the (apparently rare) approach taken by her physiotherapist “But that she looks at my physiotherapy holistically … she always asks how I’m doing, she really asks, and this morning she said I should show her how I get out of bed and back in again” (F2). Interviewees often discussed the importance of having interesting or meaningful conversations with professionals (e.g., physiotherapist or home care), and also highlighted when this relationship or obtaining support for tasks has been problematic for them due to language barriers or lack of continuity.

##### Use of care organizations (formal care)

The survey question about current use of care organizations had the largest proportion of missing data, as 56% of participants did not answer; however, we assume this is because there was not an answer option available if no organizations were needed. There were 44% of survey participants using at least one organization in 2018, and of those, most (77%) reported only using one. An interviewee recognized the unique situation they are in to be aging in Switzerland, “we in Switzerland are actually very well provided for with these organizations and associations” (F2). Yet, another recognized that even a good system can be improved. Private help (self-payment) was most commonly used (47%) in 2018 for those indicating both current and future organizational use, although slightly less preferred in future (43%). There was an increase in responses for almost all care organizations, with non-profit aid (e.g., home care support) more than doubling in value, and still anticipated by almost all (88%) who did not respond about current organization use. Interviewees described many formal caregivers from organizations who were involved in their care, providing support with basic and instrumental activities of daily living, as well as household services or taking them out for a walk. Interviewees discussed how they can also count on these care organizations, and their dependence on these organizations, as one described they need accompaniment now to go out for a walk, “so I walk with this bodyguard” (M4). Two of the interviewees brought up challenges during the initial phase of receiving support from care organizations, however expressed that these problems improved over time.

##### Multi-disciplinary team

While there was no survey data on the remaining topics, additional integrated care concepts were touched on during the interviews. There was no emergence of strong themes in this section; however, the occasional references made to these concepts shed light on integrated care. When asked about cooperation between providers, one interviewee could imagine challenges with providers trying to cooperate together “there only has to be a family doctor who has no time and says: ‘Who pays me? What’s going on? Now I have to go out to the house’. … ‘and then we have scheduling difficulties, and who pays for that?’” (F2). Still in relation to cooperation, one imagined that their professionals communicated and two felt there was good cooperation between their home care and their GP. With respect to coordination, interviewees could envision the benefits of bringing all providers together to discuss planning their care, “I could even invite the priest, the oncologist and the psychiatrist … and of course the doctor and home care. That they come and that this [future care] is managed” (F2).

##### Named coordinator

Esser et al. (2022) also determined that many interviewees felt no one, except for themselves perhaps, had an overview of their situation [40]. While some individuals could imagine this to be beneficial, many were still capable of booking their appointments by themselves or in collaboration with professionals or family members. One gave an example of a care coordination office offered by their home care support provider. Interviewees each mentioned someone different who initially organized their home care support, varying from a family member, their GP and a hospital social worker. In comparison to fragmented care one interviewee experienced in the hospital, she provided an analogy of how an architect builds something to describe how she could imagine coordinated care planning:

“...when he had to build something, that he always had the carpenter, the electrician, the plumber, the bricklayer, all at one table. And he said: ‘So, how is it going? Scheduling: you are dependent on that, he is dependent on that’. This is the only way to get to their goal” (F2).

#### Leadership and governance domain of the SELFIE framework

##### Individualized care planning

One interviewee discussed an interest in having a transparent discussion about care with all stakeholders: “questions come, ‘nursing home? In need of care? Can the relatives still handle it?’. I think that should be discussed transparently. And it should be done at a round table” (F2).

## Discussion

Given the pressing need to organize community-based care and support which helps frail older adults continue to live at home [59, 60], this study aimed to first understand their current and anticipated health and social care and support in context [61], using a population survey and interviews from the INSPIRE parent study. The synthesized data indicates home-dwelling frail older adults reported being supported from various formal and informal caregivers. Nevertheless, it is concerning that there remains a small subset of this population who have unmet needs. This is especially true given that the population of older adults in this Canton and Switzerland overall is anticipated to increase [62, 63], along with the demand on formal and informal care as dependency increases [8, 9]. When combining our current findings with existing literature, it points to focal elements (e.g., assessment of needs, named coordinator) which merit further attention to optimize integrated care [28], benefiting frail older adults, the system and reducing potential burden on informal carers.

Most home-dwelling frail older adults in our study appeared to receive support which met their perceived needs. It should be noted that this was a subjective self-reported assessment of needs which we did not further evaluate objectively in this study, and that the question also did not differentiate between health versus social support, nor whether they deemed this support as satisfactory (i.e., their qualitative perception) versus sufficient (i.e., an adequate amount) [33]. This low rate of unmet needs may be attributed to the reported formal and informal care which also helps them pursue their goal to live independently at home. There is an abundance of care organizations and services available for older adults in this Canton which is consistent with Switzerland generally [64] – a country renowned for its good health care system [65]. We postulate that this has contributed to why less than one-tenth of our survey participants perceived a need for more care and support, even when facing frailty. However, the 6.5% of frail older adults with unmet needs are of major concern. When comparing this to a study in the Netherlands, a much higher percent of home-based frail older adults in their survey reported more need for support, albeit their study included a smaller sample and a different measure of frailty [5]. Our results also showed that there are several frail older adults who are not using care organizations or services, suggesting that they are less dependent. A fraction of frail older adults themselves were even providing help to others, a finding that has been observed before in Switzerland and believed to “be a good indicator of people’s health” [66] and helps maintain their “sense of independence” [33]*.* This finding also supports the notion of maintaining a strengths-based approach to frailty by also considering the strengths/resources of older adults living with frailty [33]. If dependence increases in our population, our findings indicate a potential increased demand on care organizations and services in future to meet the needs of frail older adults. Therefore, to avoid duplication of services and fragmentation in future, this implies that coordination and communication should become progressively important for them to function together effectively as a care network and also meet the needs of the frail older adult [26, 28]. Use of information and communications technology (ICT) could help support care coordination [28, 67].

The synthesis of our findings support insight into the dynamics with involving informal caregivers in frail older adults’ care. Some frail older adults reported reliance on their sources of informal care. However, despite increased need, others did not intend to use informal care in the future, due to a fear of burdening informal caregivers, as expressed by interviewees. Our finding corroborated with existing literature which has frequently reported older adults’ fear of burdening informal caregivers or asking for help [5, 68–70]. Given the diverse roles of informal caregivers noted in our study and the expected demand in future, coupled with the well-known concern that informal caregivers are at risk of burden [10, 28], informal carers - especially relatives - are an important target. It is imperative to intervene as caregiving can impact them in many ways, such as financially, emotionally and psychologically [71, 72]. We therefore support Ambugo et al.’s (2021) suggestion that informal caregivers’ needs should also be assessed when assessing the needs of frail older adults, and connected with support [73].

Informal caregivers have an important role from an integrated care perspective in contributing to the care planning and shared-decision making processes for older adults with multimorbidity and/or frailty [28, 74, 75]. Furthermore, integrated care may positively impact informal caregivers of frail older adults [76]. Some of the burdensome responsibilities of informal caregivers could be alleviated through professionals in the system, for example by the Information and Advice Center (IAC) in the INSPIRE parent study, through helping them to find appropriate services within the system [71]. There needs to be a balance between following best practices with involving informal caregivers, yet ensuring frail older adults’ preferences are taken into consideration for the level and timing of involvement, and the type of information shared with the informal caregiver. This points to a consideration that professionals or coordinators providing care to frail older adults should be aware of and consider how to navigate and manage.

Our qualitative results regarding integrated care concepts were quite heterogenous and did not generate strong themes. For example, care coordination was a topic which seemed to be of greater familiarity to the researchers [40]. Nevertheless, the findings raised a few concerning points from an integrated care perspective, such as suggesting that the needs of home-dwelling frail older adults are not consistently evaluated, yet two interviewees could imagine that someone assessing their needs would be helpful. Integrated care guidance for older adults confirms that a comprehensive assessment of health and social needs is a key first step in the intervention of older adults with frailty [75]. Further, this assessment can help to identify priority conditions associated with declining intrinsic capacity, the type of care needed, and lead to creation of a care plan which is coordinated and tailored to their needs [28, 75, 77]. Aside from assessing their original needs, the literature recommends a key person has an overview of their situation, or that there is one consistent point of contact who manages referrals and coordinates care, among other duties [28, 67, 78]. Consistent with our quantitative findings, our qualitative data indicated these are relevant gaps in frail older adults’ care, from our view as health researchers. Informal caregivers have also suggested that they would rather turn to one person to arrange care [71, 79].

Our study largely confirms what is existing in the literature on these topics, but brought more light to the power of understanding each individual older adults’ care situation in context, which is fostered by person-centred integrated care. These results have meaning on a local level for the IAC, and could also be applied to similar community-based services aiming to assess older adults’ needs and support care coordination and integration, if appropriate in context. In the context of our research where the care law has requirements for the IAC (e.g., to include a specialist nurse to assess needs), the IAC staff will also need to collaborate well with the many formal and informal carers involved, could maintain an overview of the frail older adults’ situation, and help to relieve the potential burden on informal caregivers. Furthermore, by identifying each individual’s formal and informal caregivers involved and understanding their roles, a named coordinator can for example “map a care network,” as a starting point for care planning discussions between the older person as well as all relevant caregivers, as suggested by Grol et al. (2020) [14]. As a next step from our research, we support building from Janse et al.’s (2018) work in aiming to capture the evolving dynamics between informal and formal care when studying integrated care of home-dwelling frail older adults [24]*.*

Moving forward, the findings from our study support that the network of all formal and informal caregivers providing health and social care could indeed be viewed as a “care convoy”, as put forward previously by Kemp et al. [23] and Lambotte et al. [33]. The “care convoy” concept acknowledges the overlap between formal and informal care, and embraces the complex and dynamic nature of care networks for this population [23, 33]. When considering the properties of these care convoys, we also observed diversity in the structure and function (e.g., receiving care, self-care, and caring for others), and that an individual’s care convoy will likely evolve in future depending on their health and social situation and needs [23, 33]. As highlighted by previous authors, given the outcomes tied to these care convoys (e.g., well-being and caregiver burden), it is important to consider the contextual influences on these care convoys, for example, societal influences on role expectations which may differ [23].

### Strengths and limitations

Given the complexity and diversity of frail older adults’ care, our study is one of the first mixed methods studies to gather a more comprehensive understanding of the types of care and support used, as well as experience and future preferences of home-dwelling frail older adults, as a precursor to implementing an integrated care model. Provided a rigorous approach is taken towards data collection and analysis during both arms of the study (as demonstrated in our work), this leveraged the strengths of both quantitative and qualitative approaches to address our research question [41, 55]. Gathering a more in-depth perspective from the interviews helped to explain and expand on the survey findings and give us a better overall picture of the contributions and care dynamics involved with frail older adults’ health and social care from formal and informal caregivers. However, our study comes with limitations which need consideration. First, as mentioned in the INSPIRE parent study, the non-random sampling strategy used for the population survey could result in biases, weakening the generalizability and transferability of our results [39]. Due to recruitment issues, perhaps related to the Covid-19 pandemic, the sample size for the interviews was limited [40] though as previously described in the methods, was deemed to be sufficient for our purposes. In the quantitative part of the study, we considered the percentage of missing data to align with the rates expected from research with our target population [80], except for the question on use of care organizations, which we assumed was a result of the answer options available. We also chose to analyze the data in a more segmented way to assess those who currently used care/support separately from those who were not currently relying on care/support, although this resulted in lower denominators for some questions. Furthermore, all interview respondents were using multiple services (due to inclusion criteria), which appeared to always include home care services, therefore not representing the segment of the frail older population with no home care support. While appropriate for our research question, this leaves uncaptured voices of those who need help and support but are not receiving it, which may also be a relevant point about our survey respondents. In addition, given that care is dynamic whereby different caregivers are relied on throughout time [81], this study is only capturing a snapshot in time. Nevertheless, it gives some insight into perspectives of our target population and points to areas which could benefit from future research. In hindsight, it would have been ideal to be able to capture more questions specific to integrated care during the INSPIRE population survey, but we were limited with the survey length in accordance with stakeholder input. The interviews provided the opportunity to expand and collect some qualitative information on topics where we were not able to collect quantitative data first. Finally, use of some formal care providers (e.g., pharmacists, dentists) were not assessed in the survey. Future research that explores care networks of frail older adults should include all possible constituents.

## Conclusions

Most frail older adults in Canton BL appear well-supported, receiving formal health and social care as well as informal care and social support from various sources. Given the anticipated demand for future care and support of home-dwelling frail older adults, we recommend that efforts are in place to prevent fragmentation between health and social care as well as formal and informal care. Further research could also explore those living with unmet needs and how integrated care models impact the dynamics within the care networks of home-dwelling frail older adults.

## Supplementary Information

**Additional file 1.** Survey questions extracted from the original INSPIRE Population Survey (translated from German) (Siqeca et al., 2021).

**Additional file 2.** INSPIRE population survey respondents and dichotomized responses by content domain.

**Additional file 3.** Example Questions from the Interview Guide used in the INSPIRE parent study (translated from German) (Esser et al., 2022).

**Additional file 4.** Joint display table integrating study findings from the INSPIRE population survey and interviews.

**Additional file 5.** Concepts related to care and support of frail older adults in 2018 and preferences for future, mapped to adapted domains of the SELFIE framework, including only respondents who indicated no current use for each question respectively, but responded for future preferences.

## Data Availability

The two sources of data used in the current study are not publicly available, however enquiries about the population survey data can be sent to the corresponding author of the manuscript by Siqeca et al. (2021). Enquiries about the interview data can be sent to the corresponding author of the manuscript by Esser et al. (2022).

## Abbreviations

BL: Basel-Landschaft
GFI: Groningen Frailty Indicator
GP: General Practitioner
INSPIRE: ImplemeNtation of a community-baSed care Program for home dwelling senIoR citizEns
IAC: Information and Advice Center
PRISMA: Program of Research to Integrate Services for the Maintenance of Autonomy
SELFIE: Sustainable intEgrated chronic care modeLs for multi-morbidity: delivery, FInancing, and performancE

## References

[1] Wiles JL, Leibing A, Guberman N, Reeve J, Allen RE (2012). The meaning of “aging in place” to older people. Gerontologist.

[2] European Commission, Directorate-General for Employment, Social Affairs and Inclusion (2014). Adequate social protection for long-term care needs in an ageing society: report jointly prepared by the social protection committee and the European Commission.

[3] Lette M, Boorsma M, Lemmens L, Stoop A, Nijpels G, Baan C (2020). Unknown makes unloved—a case study on improving integrated health and social care in the Netherlands using a participatory approach. Health Soc Care Community.

[4] World Health Organization (2019). Integrated care for older people (ICOPE) implementation framework: guidance for systems and services.

[5] Verver D, Merten H, Robben P, Wagner C (2018). Care and support for older adults in the Netherlands living independently. Health Soc Care Community.

[6] Danilovich MK, Diaz L, Ciolinio JD, Corcos DM (2017). Functional resistance activities to impact frailty: a protocol for a randomized controlled trial involving home care aide and frail older adult dyads. Contemp Clin Trials Commun.

[7] Vetrano DL, Palmer K, Marengoni A, Marzetti E, Lattanzio F, Roller-Wirnsberger R (2019). Frailty and multimorbidity: a systematic review and meta-analysis. J Gerontol A Biol Sci Med Sci.

[8] Lambotte D, De Donder L, Van Regenmortel S, Fret B, Dury S, Smetcoren A (2018). Frailty differences in older adults’ use of informal and formal care. Arch Gerontol Geriatr.

[9] Jacobs M, Van Tilburg T, Groenewegen P, Broese Van Groenou M (2016). Linkages between informal and formal care-givers in home-care networks of frail older adults. Ageing Soc.

[10] Li J, Song Y, Gu D, Dupre ME (2019). Formal and informal care. Encyclopedia of gerontology and population aging.

[11] Parry C, Coleman EA, Smith JD, Frank J, Kramer AM (2003). The care transitions intervention: a patient-centered approach to ensuring effective transfers between sites of geriatric care. Home Health Care Serv Q.

[12] Sadler E, Potterton V, Anderson R, Khadjesari Z, Sheehan K, Butt F (2019). Service user, carer and provider perspectives on integrated care for older people with frailty, and factors perceived to facilitate and hinder implementation: a systematic review and narrative synthesis. PLoS One.

[13] World Health Organization (2015). World report on ageing and health.

[14] Grol SM, Molleman GRM, Wensing M, Kuijpers A, Scholte JK, van den Muijsenbergh MTC (2020). Professional care networks of frail older people: an explorative survey study from the patient perspective. Int J Integr Care.

[15] Balard F, Gely-Nargeot MC, Corvol A, Saint-Jean O, Somme D (2016). Case management for the elderly with complex needs: cross-linking the views of their role held by elderly people, their informal caregivers and the case managers. BMC Health Serv Res.

[16] SUSTAIN Consortium. SUSTAIN. Sustainable tailored integrated care for older people in Europe. SUSTAIN. 2019; https://www.sustain-eu.org/wp-content/uploads/sites/4/2019/03/SUSTAIN-Roadmap.pdf. Accessed 3 Jun 2021.

[17] Lohr KN, Institute of Medicine (US) Committee to Design a Strategy for Quality Review and Assurance in Medicare (1990). Health, health care, and quality of care. in medicare: a strategy for quality assurance: volume 1.

[18] NHS. Working in social care. NHS. https://www.healthcareers.nhs.uk/working-health/working-social-care/working-social-care. Accessed 13 Mar 2022.

[19] Timonen V. Toward an integrative theory of care: formal and informal intersections. In: Mancini JA, Roberto KA, editors. Pathways of human development: explorations of change. Lanham: Lexington Books/Rowman & Littlefield; 2009. p. 307–26.

[20] Araujo de Carvalho I, Epping-Jordan J, Pot AM, Kelley E, Toro N, Thiyagarajan JA (2017). Organizing integrated health-care services to meet older people's needs. Bull World Health Organ.

[21] National Academy of Sciences. Integrating social care into the delivery of health care: moving upstream to improve the nation’s health. Natl Acad Sci. 2019; https://www.nap.edu/resource/25467/09252019Social_Care_recommendations.pdf. Accessed 10 Apr 2020. 31940159

[22] Leutz WN (1999). Five laws for integrating medical and social services: lessons from the United States and the United Kingdom. Milbank Q.

[23] Kemp CL, Ball MM, Perkins MM (2013). Convoys of care: theorizing intersections of formal and informal care. J Aging Stud.

[24] Janse B, Huijsman R, Looman WM, Fabbricotti IN (2018). Formal and informal care for community-dwelling frail elderly people over time: a comparison of integrated and usual care in the Netherlands. Health Soc Care Community.

[25] Rudnytskyi I, Wagner J (2019). Drivers of old-age dependence and long-term care usage in Switzerland—a structural equation model approach. Risks.

[26] Broese van Groenou M, Jacobs M, Zwart-Olde I, Deeg DJ (2016). Mixed care networks of community-dwelling older adults with physical health impairments in the Netherlands. Health Soc Care Community.

[27] Leichsenring K, Billings J, Nies H (2013). Long-term care in Europe: improving policy and practice.

[28] Leijten FRM, Struckmann V, van Ginneken E, Czypionka T, Kraus M, Reiss M (2018). The SELFIE framework for integrated care for multi-morbidity: development and description. Health Policy.

[29] Bunn F, Goodman C, Russell B, Wilson P, Manthorpe J, Rait G (2018). Supporting shared decision making for older people with multiple health and social care needs: a realist synthesis. BMC Geriatr.

[30] Fast J, Keating N, Otfinowski P, Derksen L (2004). Characteristics of family/friend care networks of frail seniors. Can J Aging.

[31] Jacobs MT, Broese van Groenou MI, de Boer AH, Deeg DJ (2014). Individual determinants of task division in older adults’ mixed care networks. Health Soc Care Community.

[32] Verver D, Merten H, Robben P, Wagner C (2015). Supervision of care networks for frail community dwelling adults aged 75 years and older: protocol of a mixed methods study. BMJ Open.

[33] Lambotte D, Smetcoren AS, Zijlstra GAR, De Lepeleire J, De Donder L, Kardol MJM. Meanings of care convoys: the structure, function, and adequacy of care networks among frail, community-dwelling older adults. 2020;30(4):583–Qual Health Res, 597. 10.1177/1049732319861934. 10.1177/1049732319861934 31303115

[34] Lambotte D, De Donder L, Van Regenmortel S, Fret B, Dury S, Smetcoren A-S (2018). Frailty differences in older adults’ use of informal and formal care. Arch Gerontol Geriatr.

[35] Vermeiren S, Vella-Azzopardi R, Beckwee D, Habbig AK, Scafoglieri A, Jansen B (2016). Frailty and the prediction of negative health outcomes: a meta-analysis. J Am Med Dir Assoc.

[36] Clegg A, Young J, Iliffe S, Rikkert MO, Rockwood K (2013). Frailty in elderly people. Lancet.

[37] Altersbetreuungs- und Pflegegesetz (APG), in SGS 941. 2018. http://bl.clex.ch/app/de/texts_of_law/941/versions/2126. Accessed 10 Dec 2019.

[38] Yip O, Huber E, Stenz S, Zullig LL, Zeller A, De Geest SM (2021). A contextual analysis and logic model for integrated care for frail older adults living at home: the INSPIRE project. Int J Integr Care.

[39] Siqeca F, Obas K, Yip O, Stenz S, Vounatsou P, Briel M (2021). The INSPIRE population survey: development, dissemination and respondent characteristics. BMC Med Res Methodol.

[40] Esser J, Yip O, Huber E, Dhaini S, De Geest S, Deschodt M, Spichiger E (2022). Accepting dependency to maintain autonomy - experiences and needs of older people: a qualitative study.

[41] Creswell JW (2014). Research Design. Qualitative, quantitative and mixed methods approaches.

[42] Thorne S (2016). Interpretive description: qualitative research for applied practice.

[43] Braun T, Grüneberg C, Thiel C (2018). German translation, cross-cultural adaptation and diagnostic test accuracy of three frailty screening tools: PRISMA-7, FRAIL scale and Groningen frailty Indicator. Z Gerontol Geriatr.

[44] Steverink N, Slaets JPJ, Schuurmans H, Van Lis M (2001). Measuring frailty: developing and testing the GFI (Groningen frailty Indicator). Gerontologist..

[45] Peters LL, Boter H, Buskens E, Slaets JP (2012). Measurement properties of the Groningen frailty Indicator in home-dwelling and institutionalized elderly people. J Am Med Dir Assoc.

[46] De Witte N, Gobbens R, De Donder L, Dury S, Buffel T, Schols J (2013). The comprehensive frailty assessment instrument: development, validity and reliability. Geriatr Nurs.

[47] Beutel ME, Brähler E, Wiltink J, Michal M, Klein EM, Jünger C (2017). Emotional and tangible social support in a German population-based sample: development and validation of the brief social support scale (BS6). PLoS One.

[48] World Health Organization (2016). Multimorbidity: technical series on safer primary care.

[49] Raîche M, Hébert R, Dubois M-F (2008). PRISMA-7: a case-finding tool to identify older adults with moderate to severe disabilities. Arch Gerontol Geriatr.

[50] Malterud K, Siersma VD, Guassora AD (2016). Sample size in qualitative interview studies: guided by information power. Qual Health Res.

[51] Guest G, MacQueen KM, Namey EE (2012). Applied thematic analysis.

[52] QSR International Pty Ltd. NVivo (Release 1.6). https://www.qsrinternational.com/nvivo-qualitative-data-analysis-software/home

[53] Guest G, MacQueen KM, Namey EE (2012). Chapter 3, themes and codes. Applied Thematic Analysis.

[54] Ruggiano N, Perry TE (2019). Conducting secondary analysis of qualitative data: should we, can we, and how?. Qual Social Work.

[55] Creswell JW (2015). A concise introduction to mixed methods research.

[56] Fetters MD, Curry LA, Creswell JW (2013). Achieving integration in mixed methods designs-principles and practices. Health Serv Res.

[57] Eakin JM, Gladstone B. “Value-adding” analysis: doing more with qualitative data. Int J Qual Methods. 2020;19. 10.1177/1609406920949333.

[58] Schweizerische Konferenz fur Sozialhilfe. Armut und Armutsgrenzen 2020. https://skos.ch/fileadmin/user_upload/skos_main/public/pdf/grundlagen_und_positionen/grundlagen_und_studien/2020_Grundlagendokument_Armutsgrenzen_SKOS_d.pdf. Accessed 10 Apr 2022.

[59] de Bruin SR, Stoop A, Billings J, Leichsenring K, Ruppe G, Tram N (2018). The SUSTAIN project: a European study on improving integrated care for older people living at home. Int J Integr Care.

[60] World Health Organization (2002). Active ageing: a policy framework.

[61] Harnett PJ, Kennelly S, Williams P (2020). A 10 step framework to implement integrated care for older persons. Ageing Int.

[62] Statistisches Amt des Kantons Basel-Landschaft: Kantonale Bevölkerungsstatistik, Altersprognose BL 2020 (Basis 2018). 2018. https://www.statistik.bl.ch/web_portal/1_10_2_1. Accessed 10 Feb 2022.

[63] Lewis C, Ollivaud P (2020). Policies for Switzerland's Ageing Society: Economic Department Working Papers No. 1600.

[64] Färber A. FairCare. https://www.zhaw.ch/en/research/research-database/project-detailview/projektid/1468/. Accessed 4 Nov 2021.

[65] OECD/WHO (2011). OECD reviews of health systems.

[66] Federal Statistics Office FSO (2018). Active ageing.

[67] Goodwin N, Dixon A, Anderson G, Wodchis W (2014). Providing integrated care for older people with complex needs. Lessons from seven international case studies.

[68] Cahill E, Lewis LM, Barg FK, Bogner HR (2009). “You don’t want to burden them”: older adults’ views on family involvement in care. J Fam Nurs.

[69] Abdi S, Spann A, Borilovic J, de Witte L, Hawley M (2019). Understanding the care and support needs of older people: a scoping review and categorisation using the WHO international classification of functioning, disability and health framework (ICF). BMC Geriatr.

[70] Dostálová V, Bártová A, Bláhová H, Holmerová I (2021). The needs of older people receiving home care: a scoping review. Aging Clin Exp Res.

[71] McGilton KS, Vellani S, Yeung L, Chishtie J, Commisso E, Ploeg J (2018). Identifying and understanding the health and social care needs of older adults with multiple chronic conditions and their caregivers: a scoping review. BMC Geriatr.

[72] Committee on Family Caregiving for Older Adults, Board on Health Care Services, Health and Medicine Division, & National Academies of Sciences, Engineering, and Medicine. The Impact of Caregiving on the Caregiver. In: Schulz R, Eden J, editors. Families caring for an aging America. Washington, DC: National Academies Press; 2016. 27905704

[73] Ambugo EA, de Bruin SR, Masana L, MacInnes J, Mateu NC, Hagen TP (2021). A cross-European study of informal carers’ needs in the context of caring for older people, and their experiences with professionals working in integrated care settings. Int J Integr Care.

[74] Boult C, Karm L, Groves C (2008). Improving chronic care: the “guided care” model. Perm J.

[75] World Health Organization (2019). Integrated care for older people (ICOPE): guidance for person-centred assessment and pathways in primary care.

[76] Janse B, Huijsman R, de Kuyper RDM, Fabbricotti IN (2014). The effects of an integrated care intervention for the frail elderly on informal caregivers: a quasi-experimental study. BMC Geriatr.

[77] Turner G, Clegg A (2014). Best practice guidelines for the management of frailty: a British geriatrics society, age UK and Royal College of general practitioners report. Age Ageing.

[78] Deschodt M, Laurent G, Cornelissen L, Yip O, Zuniga F, Denhaerynck K (2020). Core components and impact of nurse-led integrated care models for home-dwelling older people: a systematic review and meta-analysis. Int J Nurs Stud.

[79] Gill A, Kuluski K, Jaakkimainen L, Naganathan G, Upshur R, Wodchis WP (2014). “Where do we go from here?” Health system frustrations expressed by patients with multimorbidity, their caregivers and family physicians. Health Policy.

[80] Hardy SE, Allore H, Studenski SA (2009). Missing data: a special challenge in aging research. J Am Geriatr Soc.

[81] Kjær AA, Siren A (2020). Formal and informal care: trajectories of home care use among Danish older adults. Ageing Soc.

