# Supplementary material for: Health and social care of home-dwelling frail older adults in Switzerland: a mixed methods study
Source: BMC Geriatr. 2022 Nov 15;22:857. doi: 10.1186/s12877-022-03552-z (PMC9663289; doi:10.1186/s12877-022-03552-z)
Supplement: Supplementary file 1 — Additional file 1. Survey questions extracted from the original INSPIRE Population Survey (translated from German) (Siqeca et al., 2021). [file 12877_2022_3552_MOESM1_ESM.pdf]

Additional File 1: Survey questions extracted from the original INSPIRE Population Survey (translated from German) (Siqeca et al., 2021)

Survey Section	Survey question	Response options
What support are you currently receiving?	From whom do you receive regular support in everyday life? *Please check all possible answers.*	Family members of the same age (e.g., spouse, partner), younger family members (e.g., children, grandchildren), friends and neighbours, I don't need
	From which organisations do you receive regular support in everyday life? *Please check all possible answers.*	Non-profit aid (e.g., Spitex [home care] support), Private help (self-payment), Alzheimer's Association, Parkinson's Association, Diabetes Association, Red Cross Baselland, Pro Senectute, Other
	What kind of help do you need or did you use in 2018? *Please check all possible answers*	I didn't need any help in 2018, Care and support at your home (public organization, private organization), Help with the housework (public organization, private organization), Meal service, Physiotherapy, Transport and assistance services (e.g., to the doctor, shopping), Day clinic, Night clinic, Old-age flat, Short stays in old people's homes/nursing homes, Other
	How often is the following support from other people available to you?* *Whether by professional people or support from family/friends*<br>a. someone who will take you to the doctor if necessary<br>b. someone who prepares food for you when you're not able to<br>c. someone to help you with your day-to-day work when you're sick<br>d. someone who can give you good advice in difficult situations<br>e. someone you can trust or talk to about personal problems<br>f. someone who understands your problems	Never, sometimes, often, always (repeated for sub-questions a-f)
	Does the support you receive in everyday life meet your needs?	Yes, I'm getting the support I need; No, I need more support
	Do you look after, care for or support another person yourself? E.g., children, elderly people, people with a disability	Yes; No
What about the support in the future?	If you become more in need of help, which people do you prefer to receive regular support from in everyday life? *Please check all possible answers*	Family members of the same age (e.g., spouse, partner), Younger family members (e.g., children, grandchildren), Friends and neighbours
	If you become more in need of help, which organizations do you prefer to receive	Non-profit aid (e.g., Spitex), Private help (self-payment), Alzheimer's Association, Parkinson's Association, Diabetes

	regular support from in everyday life? *Please check all possible answers*	Association, Red Cross Baselland, Pro Senectute, Other
	If you become more in need of help, which of the following services would you consider? *Please check all possible answers*	Care and support at your home (public organization, private organization), Help with the housework (public organization, private organization), Meal service, Physiotherapy, Transport and assistance services (e.g., to the doctor, shopping), Day clinic, Night clinic, Old-age flat, Short stays in nursing homes, Don't know, Other
How often did you need medical help in 2018?	How many times did you visit your family doctor in 2018?	__ times; or more than 10
	How often did you visit a specialist in 2018?	__ times; or more than 10
	What other medical services did you use in 2018?	*Open-ended question*

Pro Senectute: a non-profit foundation serving older adults; * = BS6 questions
