# Supplementary material for: Health and social care of home-dwelling frail older adults in Switzerland: a mixed methods study
Source: BMC Geriatr. 2022 Nov 15;22:857. doi: 10.1186/s12877-022-03552-z (PMC9663289; doi:10.1186/s12877-022-03552-z)
Supplement: Supplementary file 2 — Additional file 2. INSPIRE population survey respondents and dichotomized responses by content domain. [file 12877_2022_3552_MOESM2_ESM.pdf]

Additional file 2: INSPIRE population survey respondents and dichotomized responses by content domain

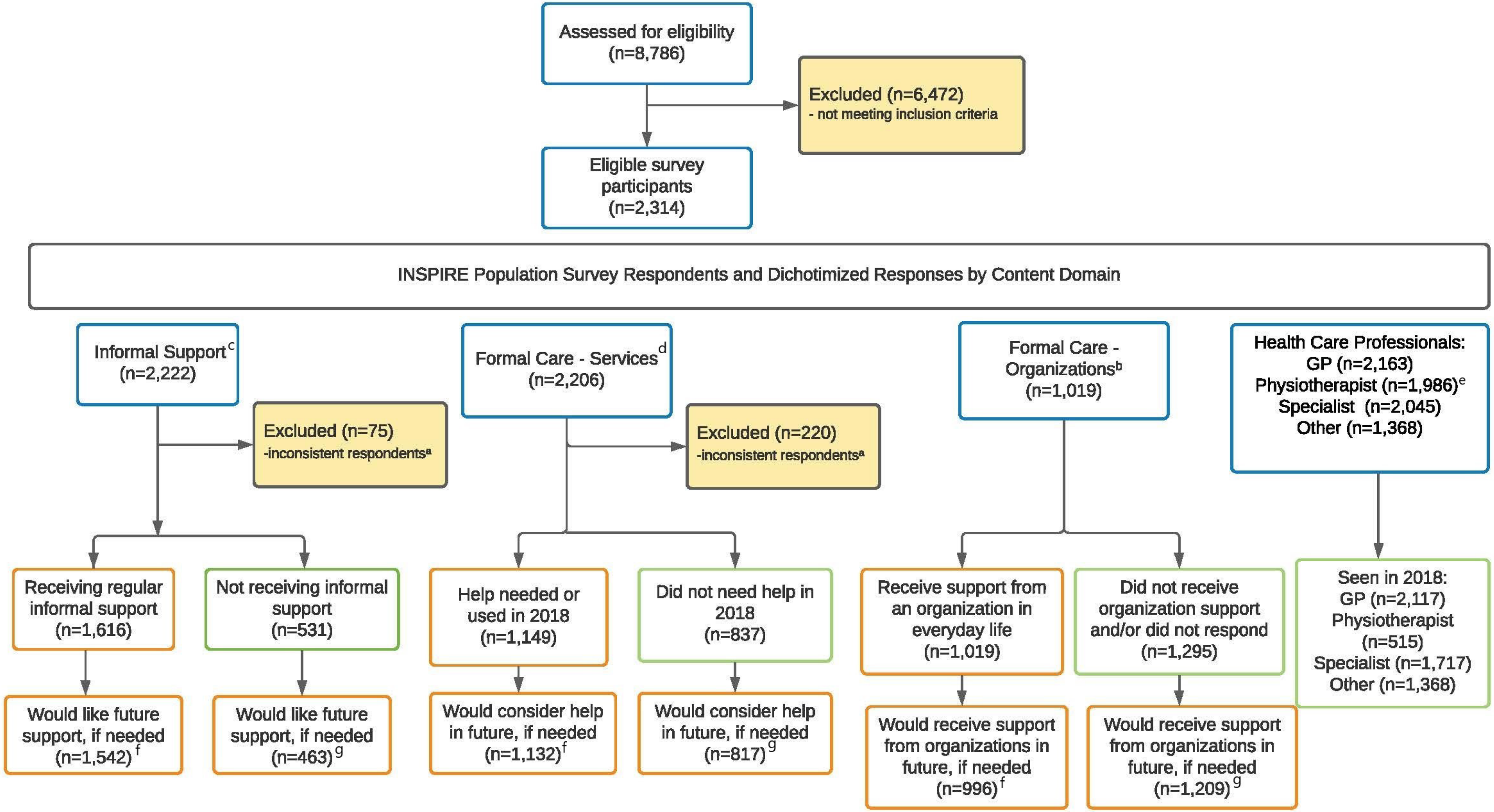

Note: There were 2,314 unique survey participants. This figure shows the number of respondents per question of each content domain on the top level (blue), and the dichotimized responses per question within each domain below (orange and green). The respondents across the domains are not unique.

[a] inconsistent respondents = individuals who provided a contradictory answer to the question
[b] the survey question about use of current formal care organizations did not provide an answer option to indicate not using any organizations
[c] missing data = 4%
[d] missing data = 5%
[e] physiotherapy values shown excluded inconsistent respondents
[f] reported in Table 2
[g] reported in Supplementary file 5
