# Supplementary material for: Health and social care of home-dwelling frail older adults in Switzerland: a mixed methods study
Source: BMC Geriatr. 2022 Nov 15;22:857. doi: 10.1186/s12877-022-03552-z (PMC9663289; doi:10.1186/s12877-022-03552-z)
Supplement: Supplementary file 3 — Additional file 3. Example Questions from the Interview Guide used in the INSPIRE parent study (translated from German) (Esser et al., 2022). [file 12877_2022_3552_MOESM3_ESM.pdf]

Additional File 3: Example Questions from the Interview Guide used in the INSPIRE parent study (translated from German) (Esser et al., 2022)

**Overview of everyday life**
• Can you tell me about the support you receive for your care, health and day-to-day needs during an ordinary week?
**Questions about the current care situation**
• Can you tell me what works well in your current care situation?<br>• Can you tell me who you contact when you have questions about your current care situation or health?
**Needs assessment**
• Can you tell me if anyone has ever looked at your situation as a whole with you and considered what kind of support you need?
**Coordination and communication**
• Is there someone who, in your opinion, keeps an eye on and manages your care as a whole?
