# Supplementary material for: Health and social care of home-dwelling frail older adults in Switzerland: a mixed methods study
Source: BMC Geriatr. 2022 Nov 15;22:857. doi: 10.1186/s12877-022-03552-z (PMC9663289; doi:10.1186/s12877-022-03552-z)
Supplement: Supplementary file 4 — Additional file 4. Joint display table integrating study findings from the INSPIRE population survey and interviews. [file 12877_2022_3552_MOESM4_ESM.pdf]

Additional file 4: Joint display table integrating study findings from the INSPIRE population survey and interviews

	INSPIRE population survey	INSPIRE interviews	
**Concept from SELFIE framework**	**Findings**	**Themes / explanation of survey finding**	**Examples of supporting illustrative quotes**
**Individual with multi-morbidity and their environment**			
Needs and preferences	• 93.5% perceived their support matches their needs	• Satisfaction with care from services/ professionals<br>• Dependence on providers/services<br>• Strong preference to stay living at home (Esser et al., 2022)<br>• Desire personal care<br>• Occasional care gaps and challenges, such as difficult relationships with providers (Esser et al., 2022)<br>• Assessment of needs (+/-)	"Tip top" (M2)<br><br>"I have to have Spitex [home care] anyway" (F3)<br><br>"So, no, I never discussed the overall situation with anyone" (M4)
Social network	• Availability of social support was lower for tangible items and higher for emotional-information items	• Receive tangible and emotional-informational social support<br>• Importance of social network	"The neighbours would also do errands for me, take care of errands, do the shopping" (F4)<br><br>"to do with rides, to do errands with and they [friends] just do me good mentally, too. ...I just know that I can turn to, so when I have them, when I also, difficulties, that I can go to them, so their help, that I can just share my thoughts. That they understand me" (F4).<br><br>[they can] "give each other courage" (F4) [when discussing with their peers about what can be improved in their situation]

Community services (formal care)	• 58% using at least 1 service (and of those, over half using 2+); 42% did not need any services<br>• Most common services used now and preferred in future:<br>o Help with the housework<br>o Care and assistance at home<br>• Large increased demand for services from now to future (e.g., meal services)	• Often described their need for support with housework and home care<br>• Commonly received support with ADLs and IADLs<br>• Transportation services took them to the hospital, a doctor or community service<br>• Provides social interaction or motivates them	"That means they clean my flat, cook for me if I want them to, take me for walks and go shopping with me. She makes my bed, so she actually does the housework for me" (M4)<br><br>"I like to talk to this woman" (M4)
**Workforce**			
Informal care	• 75% had at least one source of informal care<br>• Over half currently use support from family members of the same age, and largest increase to future was for younger family members<br>• Those not using informal care now most commonly prefer younger family members in future.	• Multiple informal caregivers involved who fulfill various roles<br>• Reliance on informal care, especially relatives<br>• Dynamics with informal caregivers<br>• Fear of burdening informal caregivers	The wife "does everything" (M3)<br><br>"You have to get up alone. You have to do it alone" (F3)<br><br>"I don't want to be the center of my family's attention with the illness" (F2)
Professionals (formal care)	• 60% had 1-6 visits with GP, 37% had 7-10+ visits<br>• 67% had 1-6 visits to specialist<br>• 30% used physiotherapy<br>• 59% had visits with other medical professionals	• Various formal providers involved serving variety of functions<br>• GP as the main contact person for questions about their health (Esser et al., 2022)<br>• Physiotherapist often involved in care - unique value<br>• Importance of relationships with professionals<br>• sometimes experienced lack of coordination between providers (Esser et al., 2022)	"If I have special questions then I discuss it with the family doctor" (M1)<br><br>"… walked into the woods with the rollator and with our feet in the leaves. And it was just beautiful for the eyes, for the taste, for the walking, that was just beautiful. And she didn't actually do much, she just made sure that nothing happened to me. Isn't that nice?" (F2).

			"And with the physio or certain other acquaintances I can still discuss other topics" (M4)
Organizations (formal care)	• 56% did not answer this question<br>• 44% using at least 1 organization<br>• If still wanting help in future, currently private help was most common (47%) but dropped for future<br>• Non-profit help doubled in value and was also most popular in future for those not receiving help currently	• Multiple organizations involved<br>• Satisfied with organizations and grateful to be in Switzerland<br>• Dependence on organizations<br>• Challenges during initial phase of receiving support from the organization, but problems improved over time.	"can also count on" [the organizations] (F2)<br><br>"we in Switzerland are actually very well provided for with these organisations and associations" (F2)<br><br>"But at the beginning, of course, it's a mad tangle (F2)"
