# Supplementary material for: Health and social care of home-dwelling frail older adults in Switzerland: a mixed methods study
Source: BMC Geriatr. 2022 Nov 15;22:857. doi: 10.1186/s12877-022-03552-z (PMC9663289; doi:10.1186/s12877-022-03552-z)
Supplement: Supplementary file 5 — Additional file 5. Concepts related to care and support of frail older adults in 2018 and preferences for future, mapped to adapted domains of the SELFIE framework, including only respondents who indicated no current use for each question respectively, but responded for future preferences. [file 12877_2022_3552_MOESM5_ESM.pdf]

Additional file 5: Concepts related to care and support of frail older adults in 2018 and preferences for future, mapped to adapted domains of the SELFIE framework, including only respondents who indicated no current use for each question respectively, but responded for future preferences

	**In Future**<br>% of respondents [a] (n)
**CORE: INDIVIDUAL WITH MULTI-MORBIDITY AND THEIR ENVIRONMENT**	
**Community Services and Transport** [b]	
Help with the housework	76% (620/817)
Care and assistance at home	86% (706/817)
Transportation and assistance services (e.g., to doctor's office, shopping)	37% (302/817)
Meal service	49% (399/817)
Elderly day care centre[c]	5% (39/817)
Apartment for older adults	25% (206/817)
Short stays in a Nursing home	14% (112/817)
Care centre with night services	1% (11/817)
Other (e.g., Hospital, cleaning)	2% (18/817)
**DOMAIN: WORKFORCE**	
**Informal caregiver support**	
Family members of the same age (e.g., spouse, partner)	44% (202/463)
Younger family members (e.g., children, grandchildren)	55% (254/463)
Friends and neighbours	43% (198/463)
**Professionals**	
Physiotherapy [b]	25% (204/817)
**Organizations** [b]	
Private help (self-payment)	25% (309/1209)
Non-profit aid (e.g., Spitex home care)	88% (1064/1209)
Pro Senectute (non profit foundation serving older adults)	30% (359/1209)
Red Cross Baselland	7% (82/1209)
Associations [d]	5% (59/1209)
Nursing home	24% (287/1209)
Other (e.g., help with cleaning)	3% (37/1209)

Note. The content domains are not mutually exclusive

[a] % of responses = The proportion of the respondents accounted for by this category due to multiple responses possible

[b] considered to be "Formal care" in this paper

[c] day care center (e.g., providing advice, support, care and integration)

[d] Associations = Combined values for Alzheimer's association, Parkinson's association and Diabetes Association
